# High Cryptic Diversity across the Global Range of the Migratory Planktonic Copepods *Pleuromamma piseki* and *P. gracilis*

**DOI:** 10.1371/journal.pone.0077011

**Published:** 2013-10-22

**Authors:** Kristin M. K. Halbert, Erica Goetze, David B. Carlon

**Affiliations:** 1 Department of Oceanography, School of Ocean and Earth Sciences and Technology, University of Hawaii at Manoa, Honolulu, Hawaii, United States of America; 2 Department of Biology, University of Hawaii at Manoa, Honolulu, Hawaii, United States of America; American University in Cairo, Egypt

## Abstract

Although holoplankton are ocean drifters and exhibit high dispersal potential, a number of studies on single species are finding highly divergent genetic clades. These cryptic species complexes are important to discover and describe, as identification of common marine species is fundamental to understanding ecosystem dynamics. Here we investigate the global diversity within *Pleuromamma piseki* and *P. gracilis*, two dominant members of the migratory zooplankton assemblage in subtropical and tropical waters worldwide. Using DNA sequence data from the mitochondrial gene cytochrome c oxidase subunit II (mtCOII) from 522 specimens collected across the Pacific, Atlantic and Indian Oceans, we discover twelve well-resolved genetically distinct clades in this species complex (Bayesian posterior probabilities >0.7; 6.3–17% genetic divergence between clades). The morphologically described species *P. piseki* and *P. gracilis* did not form monophyletic groups, rather they were distributed throughout the phylogeny and sometimes co-occurred within well-resolved clades: this result suggests that morphological characters currently used for taxonomic identification of *P. gracilis* and *P. piseki* may be inaccurate as indicators of species’ boundaries. Cryptic clades within the species complex ranged from being common to rare, and from cosmopolitan to highly restricted in distribution across the global ocean. These novel lineages appear to be ecologically divergent, with distinct biogeographic distributions across varied pelagic habitats. We hypothesize that these mtDNA lineages are distinct species and suggest that resolving their systematic status is important, given the ecological significance of the genus *Pleuromamma* in subtropical-tropical waters worldwide.

## Introduction

Plankton are by definition weak swimmers, and their horizontal movement in the ocean is largely controlled by ocean circulation and current structure. Not surprisingly, holoplanktonic organisms therefore are often expected to experience high gene flow among populations, and exhibit little to no genetic differentiation across their distributional range [1]–[3]. However, recent molecular studies on holoplankton are finding that many nominal species are composed of a number of genetically divergent clades with highly similar morphology, with examples now reported from nearly all pelagic metazoan phyla as well as a number of eukaryotic phytoplankton and protistan groups (e.g., chaetognaths, [4], [5]; copepods, [6]–[8]; pteropod molluscs, [9], [10]; cnidarians, [11], [12]; coccolithophores, [13]; diatoms, [14]; foraminifera, [15]–[17]). In some cases, these studies are finding that species initially described to be cosmopolitan in distribution, with ranges spanning multiple ocean basins, in fact consist of a mosaic of evolutionarily divergent populations that are restricted in distribution to particular pelagic habitats. For example, the common planktonic copepod *Rhincalanus nasutus* was once thought to be cosmopolitan in broadly eutrophic waters worldwide [18]–[20], but is now well-understood to consist of a complex of species, with many lineages occurring in only a single coastal upwelling ecosystem [6], [21]. Such observations challenge the common perception of zooplankton as highly dispersing species that are genetically connected throughout widespread oceanic distributions [22], and emphasize that regional, rather than basin-scale, oceanographic processes may be the primary drivers for these populations.

Although relatively few prior studies have fully characterized zooplankton cryptic species complexes, results to date suggest that cryptic species can be recently or deeply divergent, occur in allopatry, parapatry or sympatry with close congeners, and there can be many or few cryptic species within a given species complex [6], [7], [12], [21], [23]–[30]. Cryptic species also can be found within species that are common in the ocean’s surface waters (e.g., this study; [23]), as well as in species that are relatively rare [28]. Because cryptic species can heavily bias our perceptions of large-scale biogeographic patterns [31], [32], it is important to characterize, where possible, the true diversity within common and ecologically important groups. Accurate knowledge of zooplankton species diversity is also fundamental to mechanistic understanding of pelagic food web structure and function, one of the primary goals of biological oceanography.

Genetic data have been central to the discovery of evolutionarily divergent lineages within zooplankton species, in particular given high morphological stasis in many copepod groups [33]–[36]. Because many of the newly discovered zooplankton lineages are very divergent genetically, species boundaries have been effectively detected using relatively few molecular markers [6], [23], [24]. Multi-locus genetic data are particularly important in cases where genetic differentiation among species is comparable to that among populations, and it is difficult to detect the transition to non-reticulating gene trees that occurs at the separation into distinct species. There also are some reported cases of deeply divergent mtDNA lineages within single species, and multi-locus data are valuable for detecting these patterns (e.g., [37], [38]). A number of studies of marine zooplankton using multiple genetic markers have found congruence and support for the inference of distinct cryptic species across different genome regions [6], [23], [30], while other studies have found broadly concordant patterns but lower resolution at nuclear markers, even in cases where observed divergence at mitochondrial markers was quite high [30], [39], [40]. In sum, although single marker datasets have significant limitations (e.g., [41]), the observation has been that mitochondrial DNA sequence markers were informative in other genetic studies of zooplankton cryptic species complexes, in part due to the fact that in these under-studied pelagic invertebrate groups, many very divergent lineages remain to be discovered and described.

Here we investigate global diversity within the nominal species *Pleuromamma piseki* and *P. gracilis*, two dominant members of the migratory zooplankton assemblage in subtropical and tropical waters worldwide. These species co-occur and are abundant in a range of pelagic ecosystems, though they have sometimes been reported to have slightly distinct oceanographic distributions (e.g., [42]–[44]. Recent work reports *P. piseki* and *P. gracilis* to occur in oceanic subtropical waters (central gyres), but *P. gracilis* also often occurs in high abundance in subtropical convergence and transitions zones (e.g., Gulf Stream extension; [43]). In the subtropical North Pacific, 76% of the animals collected in the euphotic zone at night are members of the genus *Pleuromamma*, with *P. piseki* and *P. gracilis* dominating the 0.5–1 mm size fraction [45], [46]. Both species are diel vertical migrators (DVM) and they have similar depth distributions, with adults in the 0–100 m layer during the nighttime and at 200 to >400 m depths during the daytime [44], [47]–[50]. As a result of this migratory behavior, these species play an important role in marine biogeochemical cycles (e.g., [51]–[53]), and actively export significant fractions of carbon and nitrogen from surface layers into the upper mesopelagic zone [45]. Both species are omnivores [54], and they feed only at night while in near-surface layers [48], [50].

The systematics of the genus *Pleuromamma* is considered well-resolved (11 species; [42], [55]), and only a single new species has been described since 1950, *P. johnsoni* [56]. In the most recent comprehensive revision of the genus, Steuer [42] described three forms within *P. gracilis* s. l.; forma minima, forma Piseki, and forma maxima. Forma Piseki was elevated to species status by Farran [57], while the names f. minima and f. maxima are not in contemporary use (e.g., [55]; f. minima considered synonymous with *P. gracilis* s. s.). Forma minima was initially described as being circumglobal in subtropical and tropical waters (Karte 14 in Steuer 1932), and f. maxima occurred predominantly, though not exclusively, in the southern transition zone of the Atlantic, Pacific and Indian Oceans. Morphological differentiation between *P. piseki*, *P. gracilis*, and the only other described small-bodied species in the genus, *P. borealis* Dahl 1893, is relatively slight [55], [58], [59], and males and juveniles of these species are often lumped in ecological studies due to difficulties in identification [44], [49]. One prior study of intraspecific morphological variation within *P. piseki* and *P. gracilis* did not find evidence of additional undescribed species ([60], N = 17, 18 individuals), but the only prior study to report genetic data for these species did find unusually high genetic divergence within *P. piseki* (N = 2 specimens; [61]).

Our initial goal was to assess the population genetic structure of *Pleuromamma piseki* across its global distribution in tropical and sub-tropical waters, in order to better understand gene flow and population connectivity in marine holoplankton. However, our initial phylogenetic results based on mitochondrial cytochrome c oxidase subunit II (mtCOII) DNA sequences revealed divergent genetic clades within this nominal species. Our next objective then was to characterize the diversity within this newly discovered cryptic species complex, and determine the phylogenetic relationship of the described species *P. piseki* and *P. gracilis* to the undescribed genetic lineages present in the group. We also sought to characterize, to the extent possible, the oceanographic distribution of all lineages in the species complex. We conducted preliminary efforts to detect morphological differentiation between genetic lineages, in order to find potential characters for species identification. And finally, due to problems with polymerase chain reaction (PCR) amplification of orthologous mtDNA gene copies, the final goal was to verify that the genes being amplified were functional mitochondrial copies of mtCOII. Here we report on the global diversity and distribution of genetic lineages within the *P. piseki – P. gracilis* species complex, based on 522 specimens collected throughout the Atlantic, Pacific, and Indian Oceans.

## Materials and Methods

### Sample Collection and Specimen Identification

**Figure 1:**
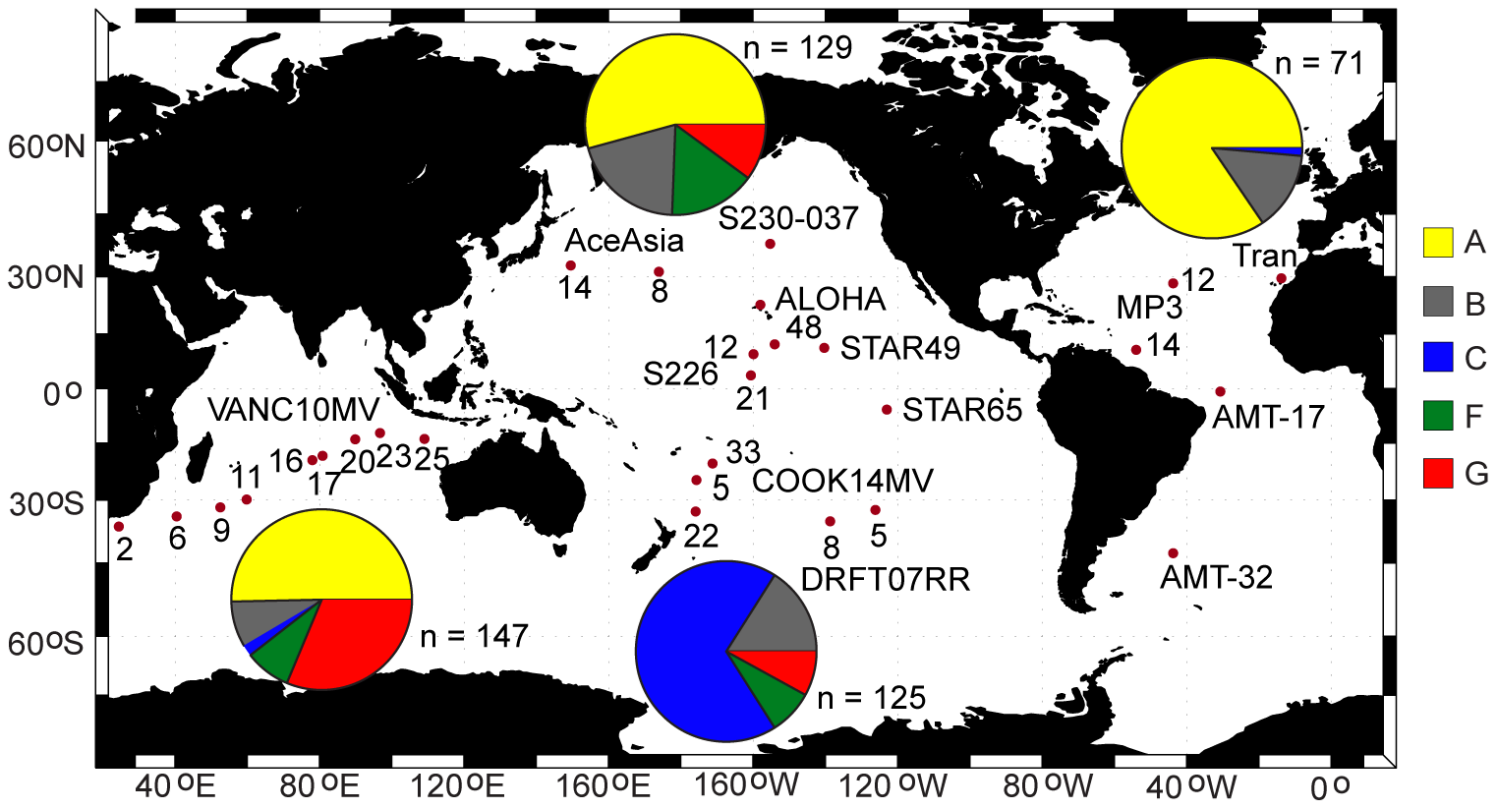
Distribution of sampling locations (red circles) and the frequency of individuals from different genetic clades in the Atlantic, Pacific, and Indian Oceans (pie charts), based on gDNA amplifications of mtCOII. Total sample sizes (n) for each ocean region are given next to the pie charts. Clade colors are defined in figure 2. The cruise and station number for sample sites are listed next to the symbols (detail in Table 1).

Zooplankton were collected in bulk plankton samples from 32 locations in the Atlantic, Pacific, and Indian Oceans (Table 1, Fig. 1). Permits were not required for these collections, and the work did not involve endangered or protected species. Sampling depths varied across cruises. Oblique tows were made on the ACE-ASIA and STAR00 cruises sampling between 200 m and the surface. Plankton tows on most other cruises sampled down to >700 m (Table 1). The majority of these samples were obtained by oblique tows of either a 0.71-m diameter bongo net or a 1-m ring net (202–333 µm mesh). One sample from the North Pacific (S230-037-TT) was collected in an oblique tow with a Tucker trawl (333 µm mesh), to a maximum depth of 530 m. Bulk plankton were preserved in 95% non-denatured ethyl alcohol, changed to new alcohol within 24 h of collection, and maintained at −20°C to minimize degradation of DNA. Specimens were sorted from bulk plankton samples in the laboratory. Only adult females were included due to the relative rarity of males in our samples, and the reported difficulty in distinguishing males of the small-bodied species in this genus.

**Table 1:** Summary of 522 specimens collected from 32 locations included in this study of the *P. piseki – P. gracilis* species complex.

Cruise	Station	Ocean	Depth	Lat	Long	Collection	Clade Counts														
		Region	(m)			Date	Total	A1	A2	B1	B2	C	D	E	F	G	H	I	J	K	L
0106 Tran	CAN 1	NA	400	29.37	−13.48	6/22/01	22	14	8												
MP3	12-06-00	NA	800	29.57	−45.03	7/2/01	19	13	6												
MP3	14-01-00	NA	700	12.04	−55.26	7/8/01	23	11	8	1		1							2		
AMT20	16	NA	300	13.45	−38.95	10/30/10	1			1											
AMT20	17	NA	300	10.57	−32.00	10/31/10	6			6											
AMT20	18	NA	300	7.81	−30.16	11/1/10	2			2											
AMT20	32	SA	200	−41.66	−45.09	11/20/10	10							10							
AMT20	33	SA	200	−44.20	−48.94	11/21/10	7				2			5							
Ace-Asia	8	NP	200	31.24	173.92	3/22/01	16	8		3			1		2					2	
Ace-Asia	14	NP	200	32.86	149.52	3/29/01	24	14		8										2	
S226	12-MN	NP	450	10.78	−161.22	12/3/09	6			1					3	2					
S226	21-MN	NP	316	5.02	−161.93	12/9/09	19						1		8	10					
S226	48-MN	NP	287	13.47	−155.32	12/26/09	21	19		2											
S230	037-TT	NP	750	38.12	−155.26	7/10/10	19	14		5											
HOT201	ZP890	NP	160	22.75	−158.00	5/29/08	9	9													
STAR00	M00-49	NP	200	12.49	−141.59	9/4/00	21	6		6	1				7	1					
STAR00	M00-65	SP	200	−4.44	−124.33	9/12/00	21			2	2	1			4	10	2				
COOK14MV	5	SP	950	−23.52	−177.05	10/7/01	20			1		14			4				1		
COOK14MV	23 & 22	SP	950	−31.59	−177.23	10/17/01	23			4		18							1		
COOK14MV	33	SP	950	−19.19	−172.54	10/24/01	24			3		19			1				1		
DRFT07RR	5	SP	910	−31.21	−127.50	12/20/01	23			3		19			1						
DRFT07RR	8	SP	950	−34.02	−140.03	12/23/01	21			5		14							2		
DRFT07RR	18	SP	1040	−40.53	−173.00	12/28/01	6							6							
VANC10MV	2	IO	920	−35.07	24.50	5/16/03	22	9	6			2			4	1					
VANC10MV	6	IO	910	−34.05	40.51	5/20/03	24	13	7			1			2				1		
VANC10MV	9	IO	950	−31.83	52.61	5/23/03	26	12	5		5			3					1		
VANC10MV	11	IO	950	−29.85	59.84	5/25/03	24	18	4						2						
VANC10MV	16	IO	950	−19.75	78.01	5/30/03	6									5					1
VANC10MV	20	IO	950	−13.96	89.94	6/5/03	24			4	2				1	17					
VANC10MV	23	IO	850	−12.22	96.79	6/7/03	17			1					3	7		6			
VANC10MV	25	IO	1070	−13.85	109.04	6/10/03	16									16					
						**Total**	**522**	**160**	**44**	**58**	**12**	**89**	**2**	**24**	**42**	**69**	**2**	**6**	**9**	**4**	**1**

**Figure 2:**
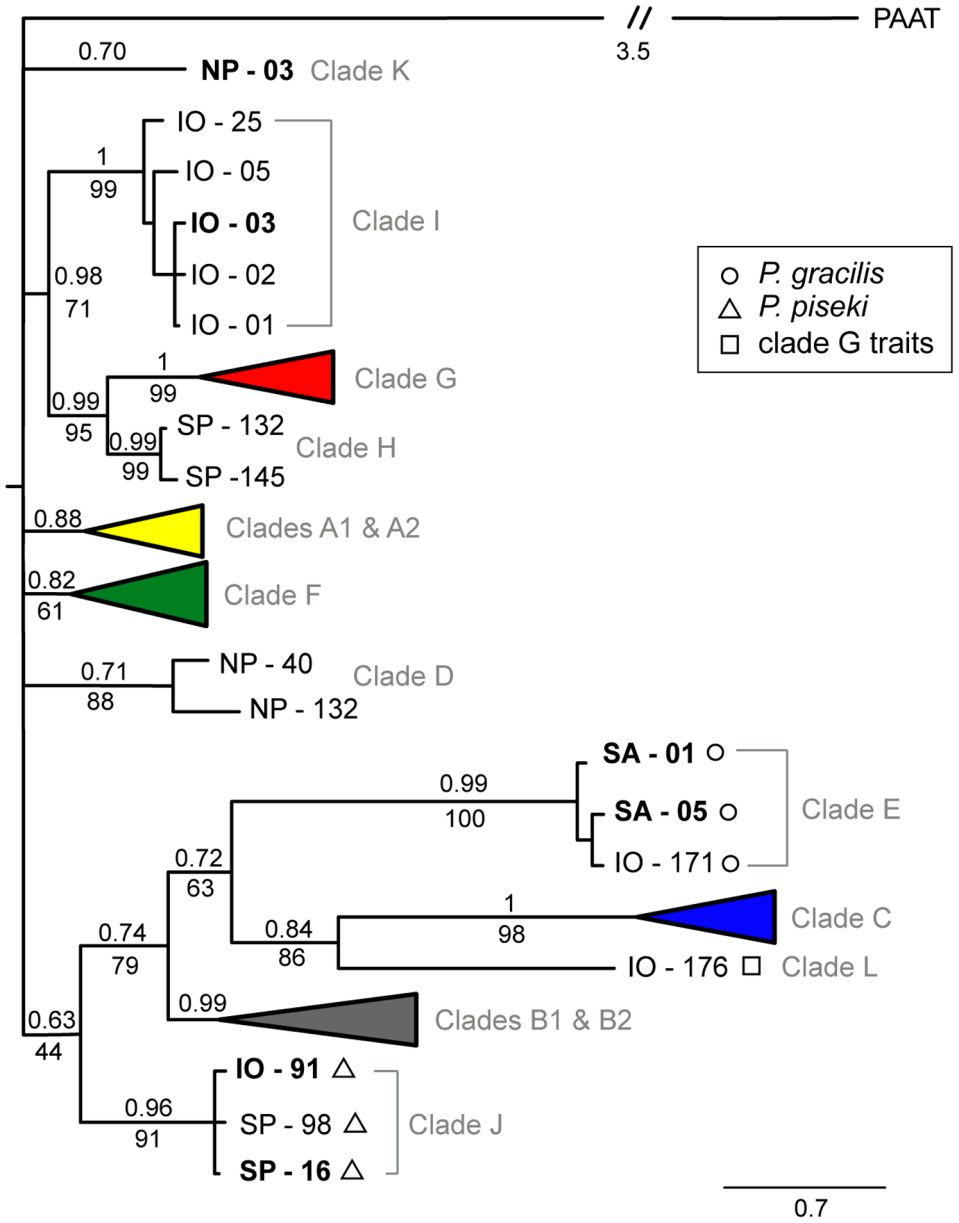
Bayesian phylogeny of 259 unique mtCOII haplotypes in the *P. piseki- P. gracilis* species complex (610-bp). For clarity, five major clades are condensed into colored triangles on the tree (color as in Fig. 1). These 5 clades are shown in detail in figures 3 & 4. Bayesian posterior probability values are given above the branches, and bootstrap values from maximum likelihood (ML) analyses are given below the branches, when that node was supported in both Bayesian and ML analyses. **Bold** text indicates haplotypes that were sampled more than once. The tree is rooted, with *Pareucalanus attenuatus* defined as the outgroup (labeled PAAT; Calanoida, Eucalanidae). The scale bar is in units of substitutions per site (0.7). Shape symbols beside the haplotype identifiers indicate morphological traits of one or more specimens of that haplotype: triangle = *P. piseki* – like characters, circle = *P. gracilis* – like characters, square = ‘clade G – like’ characters (see *Results* for more detail). Morphological trait observations for major clades A, B, C, F, G are shown in figures 3 & 4.

The numbers of specimens (N) from each station are categorized by clade identification, as shown in figure 2. NA = North Atlantic, SA = South Atlantic, NP = North Pacific, SP = South Pacific, IO = Indian Ocean. Depth is maximum depth of tow (m).

This study initially focused on *Pleuromamma piseki*. This species is characterized by relatively small body size (1.7–2 mm adult females), a pigment spot on the right lateral prosome, adult females that have a broad black pigmented area around the copulatory pore, a marked groove on the left side of the genital double-somite close to the posterior margin, and the anal somite is parallel-sided in dorsal view [55], [57], [62]. However, after our initial genetic results indicated a number of genetically divergent clades within *P. piseki*, we broadened the taxonomic coverage of the study to include *Pleuromamma gracilis*. Specimens of *P. gracilis* (N = 23) from three regions in the Indian (VANC10MV-09), North Atlantic (AMT20-16, 17, 18), and North Pacific Ocean (S226-12-MN, Table 1) were included that appeared to have classic morphological traits for *P. gracilis* (‘*gracilis*-like’). The black, pigmented area around the copulatory pore was small in these specimens, in comparison to *P. piseki*, and located near the distal end of the genital boss. *Pleuromamma gracilis* also lacks the marked groove on the left side of the genital double-somite that is parallel and close to the posterior margin and body size is slightly smaller in *P. gracilis* than in *P. piseki*. Although common in our material, *P. borealis* was not included, given our initial focus on *P. piseki*. In expanding the taxonomic coverage of the study, we noticed that morphological variation among small-bodied *Pleuromamma* specimens was higher than described in the prior literature. Therefore, we made observations on morphological characters in 199 specimens prior to DNA extraction, including measurements of body size, variation in the shape of the genital double-somite in lateral view, the shape and location of the black genital pigment spot, and the presence of a groove on the left side of the genital somite (not all characters were recorded for all specimens). The prosome length (PL) was measured for all specimens in right lateral view, and the total length (TL) was measured on straight-bodied individuals. We also recorded the location of the pigment spot on the prosome (right/left), in order to assess for any variation in this trait (previously reported at low frequency in *P. piseki*, [42], [63]). Digital images and paired mtCOII DNA sequence data were obtained from 18 specimens collected at three locations (DRFT07RR-18, AMT20-33, VANC10MV-16), for preliminary examination of morphological traits that differentiate genetic clades. Images of specimens were also taken from two additional sampling sites, S226-48MN and COOK14MV-33, but these specimens have no paired DNA sequence data. Clade assignment for these specimens is based on available mtCOII data from other specimens from the same location (clades A and C, clades defined in Fig. 2). Digital images of the posterior prosome and urosome were taken with a Leica MZ9.5 stereomicroscope and SPOT Insight Mozaic camera, with animals oriented in dorsal, ventral and right lateral views. Morphological observations reported here are preliminary, and are included in order to indicate characters that may prove informative in future systematic studies of this group. We tested for median differences in prosome length (PL) between genetic clades using a Kruskal-Wallis one-way analysis of variance (ANOVA; by ranks) followed by multiple comparison procedures (Dunn’s methods). A total of eight clades with six or more measured specimens each were included in this analysis (clades A, B, C, E, F, G, I, J). Clades A1 and A2, and B1 and B2 were combined.

### DNA Extraction and PCR Amplification

Genomic DNA (gDNA) was isolated from individual adult female copepods using the DNeasy Blood and Tissue kit (Qiagen). The manufacturer’s protocol was followed, with the following modifications: the 55°C lysis incubation step was decreased to 1 hr, the ATL buffer and proteinase K volumes were reduced by half, to 90 µl and 10 µl respectively, and the elution incubation step was lengthened to 10 min.

The mitochondrial gene cytochrome oxidase subunit II (mtCOII) was chosen as the primary genetic marker for this study. For initial mtCOII primer design, an alignment was created that included mtCOII data from *E. bungii* [64] as well as *Pareucalanus attenuatus, Euchaeta rimana*, and *Haloptilus longicornis* (Halbert & Goetze unpub). One set of universal primers, COIIF3 [5′– TTT GGT CTA CAG GAT GCT AAC TC –3′] and COIIR3 [5′– GGG ACT ATA TAG GCA TCA AAC TC –3′], were developed that amplified a 326-bp fragment of mtCOII. Using this initial sequence, a species-specific primer, PLPICOIIFC [5′– ATT AAC TAT TAA AGC GCT TGG –3′], was designed to amplify the remainder of the mtCOII gene (401 bp), 159-bp of a non-coding intergenic region, adenosine triphosphate synthase subunit 8 (ATP8, 164-bp), and 169-bp of adenosine triphosphate synthase subunit 6 (ATP6). Using these data, the species-specific primer PLPIATP8R1 [5′– ACC ACA AGC CAG TTT ATT GGT GA –3′] was designed and used with PLPICOIIFC for routine amplification of a 558–583-bp mitochondrial fragment consisting of the following genes (from 5-3′): 401-bp of COII, the intergenic region, and 12-bp of ATP8. We obtained sequence data for this region from a total of 522 specimens. This primer set amplified a single-sized product in the majority of specimens, however for ∼ 7% of specimens, double-banded products where visualized on agarose gels (not sequenced). In order to determine clade membership of these specimens, primers PLPICOIIFC and COIIR10 (5′ – TCC TCA TGT ATA CAA ATT ACT CAA –3′) were used to amplify mtCOII in eight animals (376-bp).

PCR amplification was performed in 20 µl reaction volumes with Bioline BIOLASE DNA polymerase (0.24 µl, initial concentration 5 U/µl), NH_4_-based Reaction Buffer (2 X), MgCl_2_ (2 mM), dNTPs (0.2 mM), forward and reverse primer (0.3 µM each), and DNA template (∼200 ng, 2.4 µl). Amplification cycles included a DNA denaturation step at 94°C for 2 min, followed by 35 cycles of 30 s denaturing at 94°C, 30 s annealing at 55°C, 1 min of extension at 72°C, with a final extension at 72°C for 4 min. PCR products were purified using shrimp alkaline phosphatase and exonuclease I (USB Corporation) for 30 min at 37°C. Sequencing reactions were performed using BigDye terminator chemistry, and were analyzed on an ABI 3730XL capillary-based sequencer. Both strands were sequenced for all specimens.

### Verifying Functional Gene Copies

Our initial genetic results indicated a number of highly divergent lineages within *Pleuromamma piseki.* Because NUMTs are common in crustaceans [65]–[67], we tested the possibility that we were including sequence from nuclear pseudogenes. To test for NUMTs, we sequenced PCR products from both genomic DNA and mRNA transcripts of mtCOII (RT-PCR). Both gDNA and RNA were isolated from 17 individual copepods from three mtDNA clades that were collected in the subtropical North Pacific (station Aloha, 22.75°N 158°W, 11/26/11), preserved in RNAlater (Sigma-Aldrich), and stored at −80°C. DNA and RNA were separated in different aqueous phases through the addition of Triazol (Sigma-Aldrich) and chloroform. DNA was precipitated with 95% EtOH, washed twice with 0.1 M sodium citrate - 10% EtOH and twice with 75% EtOH, and resuspended in 8 mM NaOH, 0.1 M HEPES. Total RNA was precipitated with isopropanol, washed with 75% DEPC-EtOH, resuspended with sodium citrate (1 mM, 6.4 pH, AMBION), and treated with DNase (TURBO DNA-free, AMBION). Reverse Transcriptase PCR (RT-PCR) was performed to synthesize cDNA from RNA using SUPERSCRIPT III (Invitrogen) following the manufacturer’s recommended protocol. We used the primer PLPICOIIRC [5′ – TTA AAA AAT CAT TTG TCC TTA CCA C –3′] for cDNA synthesis. The reaction was incubated at 55°C for 50 min, followed by 70°C for 15 min. Complementary DNA synthesized from RNA extractions were PCR amplified for mtCOII using primers PLPICOIIFE [5′ – CCC TAT TAT GGA AGA GCT AA –3′] and PLPICOIIRC, both of which are located within the coding region of mtCOII. Genomic DNA extractions were amplified using primers PLPICOIIFC and PLPIATP8R1 for the entire mtCOII – ATP8 region, including both coding and non-coding regions; this is the same fragment used for gDNA amplifications in all other specimens. PCR also was conducted on extracted RNA in order to verify the absence of contaminating gDNA in these samples. The same PCR cycling conditions were used as described above. Finally, we also chose three individuals in which we clearly amplified different sized fragments, and cloned and sequenced the PCR products in order to characterize potential NUMTs (see Text S1).

### Sequence Data Analyses and Phylogenetics

Sequences were edited using Geneious (v.5.1.6, Biomatters), and base calls were confirmed by aligning both strands. To assess phylogenetic relationships among specimens, maximum likelihood (ML) and Bayesian trees were inferred from 259 unique haplotypes that appeared to be functional gene copies based on the absence of premature stop codons in the amino acid translation. ML analyses were conducted using RAxML v.7.2.8 [68] and the general time reversible (GTR) model of nucleotide substitutions with the invariant (I) model of rate heterogeneity was selected as the best-fit model for the data (using *MEGA* version 5; [69]). The data set was partitioned into two regions: mtCOII (396-bp) and the intergenic spacer +7-bp of ATP8 (152 to 177 bp), in order to allow substitution rates to be estimated and optimized for each partition individually. Nodal support was assessed by bootstrapping across nucleotide sites with 1000 replicates. Bayesian trees were inferred with MrBayes v.3.2.0 [70], [71]. We selected substitution models for the different partitions using the Akaike information criterion corrected for finite sample sizes (AICc) as implemented in *MEGA*5 [69]. The best fitting model was found to be GTR with gamma (G) distributed rates for the first partition and GTR+G+I for the second partition. Four independent chains were run with a heating parameter of 0.2, and 25% of trees were discarded for the burn in. Clades with bootstrap support or Bayesian posterior probabilities of >70% were considered well supported. A second Bayesian tree was inferred that included cDNA sequences that are known to be functional. The purpose of this tree was to verify clades that are real taxonomic entities, by comparing the placement of cDNA and gDNA sequences. All trees were rooted with *Pareucalanus attenuatus* (Calanoida, Eucalanidae). To quantify genetic distances between clades, the evolutionary divergence over sequence pairs between and within clades were averaged using the Kimura 2-parameter model (in *MEGA*5). Evolutionary divergence values are reported as the percentage of base substitutions per site, averaged over all sequences pairs within/between clades.

### Geographic Distribution and Structure

Global distribution patterns of *P. piseki – P. gracilis* genetic clades were examined in a number of ways. First, we mapped the distribution of all clades in the global ocean by plotting the presence/absence of each genetic clade for each collection site (using m_map, Matlab v. 7.14). These plots give initial insights into the biogeographic distribution of these genetic populations, but are preliminary, particularly in ocean regions where clades were absent and sampling coverage was low. Second, a contingency analysis was conducted to statistically test for non-random spatial structure in the frequency of genetic clades across all oceans (using R v.2.10.1). Clades A, B, C, F, and G were included, and geographic regions were categorized as Indian, North Pacific, South Pacific, North Atlantic, and South Atlantic Ocean.

Finally, we examined the population structure within clade A across the Indian, Pacific, and Atlantic Oceans, given the cosmopolitan distribution and higher sample size for this clade (total N = 204). We used the estimator θ_ST_ to make pairwise comparisons among our 13 localities and an analysis of molecular variation (AMOVA, [72]) to quantify the amount of genetic variation partitioned between different levels of hierarchical subdivision. Populations were grouped by ocean basin: Indian Ocean - four stations, North Pacific Ocean – six stations, North Atlantic Ocean – three stations. The number of permutations used for hypothesis testing was 20,000. Analyses were conducted using Arlequin v3.5.1.3 [73]. Q-values [74] were determined for all P-values <0.05 to determine the false discovery rate (FDR) in tables of pairwise comparisons. To calculate Q-values, we used the software Q-VALUE written in R and available from John Storey (http://genomics.princeton.edu/storeylab/qvalue/).

## Results

### Sequence Data and Descriptive Statistics

A total of 522 specimens from 32 locations were successfully sequenced for the targeted mtDNA region using primers PLPICOIIFC and PLPIATP8R (Table 1). The fragment length ranged between 558 and 583 bp, with an average of 570 bp, due to the varying length of the intergenic region. In total, we sampled 268 unique haplotypes with 214 singletons and 54 haplotypes occurring more than once in the global data set. The number of haplotypes occurring in a single location ranged from 2 to 19, with an average of 12.5 haplotypes. An alignment that included 399-bp of mtCOII, the intergenic region, and 7 bp of mtATP8 was used in phylogenetic analyses (putative functional copies). Within the final alignment, there were 262 variable sites; 52 of these were single substitutions and 207 were parsimony-informative. The number of synonymous and non-synonymous nucleotide substitutions was 380 and 75, respectively. We also generated an alignment including cDNA sequences, and the final length of this alignment was 612-bp (data are available under GenBank accession numbers KF006539– KF006807).

### Phylogenetic Trees – Deep Mitochondrial Clades

**Figure 3:**
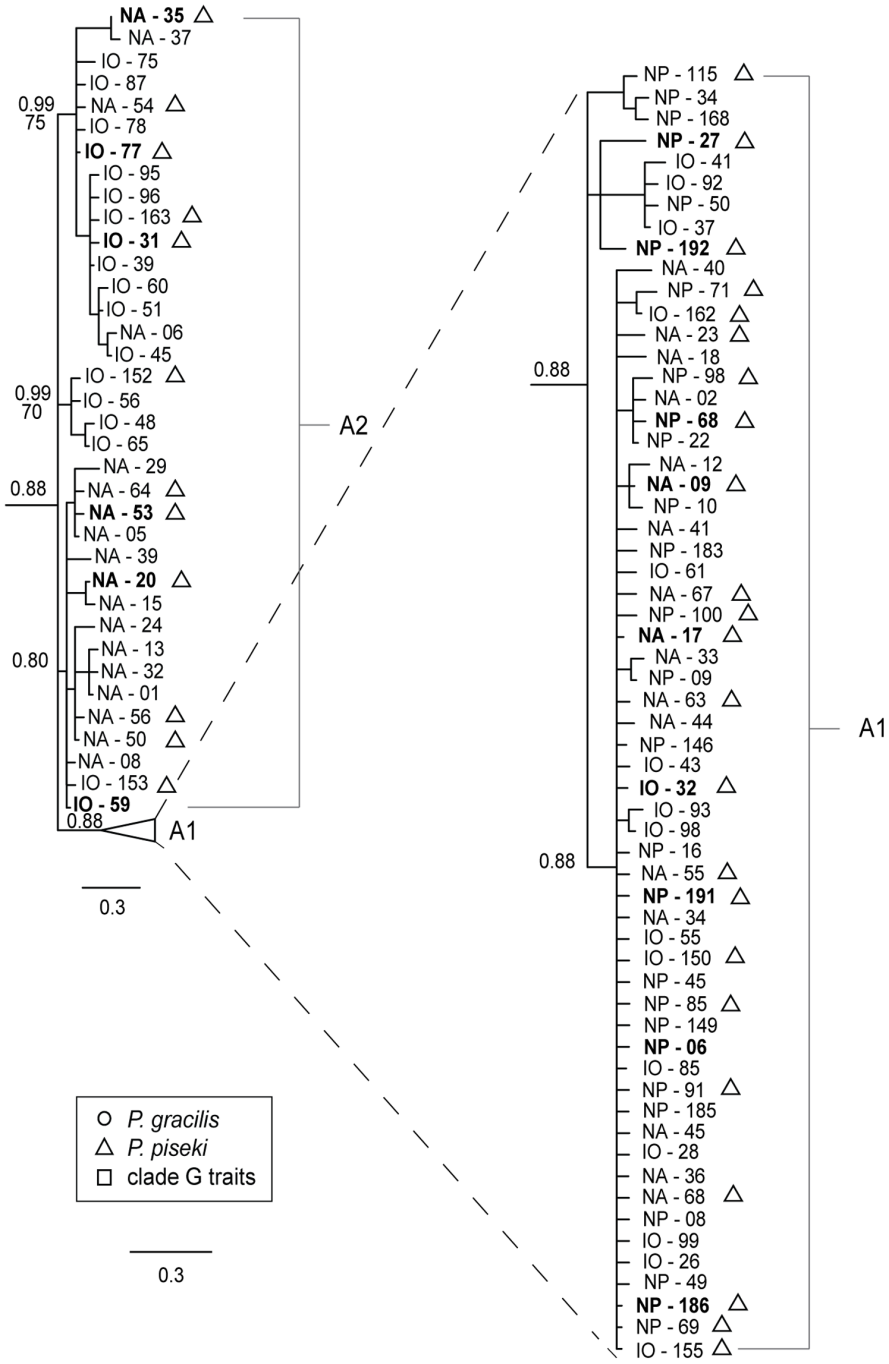
Bayesian mtCOII phylogeny of *P. piseki – P. gracilis* clade A, with sub-groups A1 and A2 as shown. Bayesian posterior probability values are given above the branches, and bootstrap values from ML analysis are given below the branches, when that node was supported in both Bayesian and ML analyses. **Bold** text indicates haplotypes that were sampled more than once. The scale bar units are in substitutions per site. Sample identifiers begin with the ocean basin that individuals were sampled from, IO = Indian Ocean, NP = North Pacific, SP = South Pacific, NA = North Atlantic, SA = South Atlantic. Shape symbols indicate morphological traits, as defined in the legend.

**Figure 4:**
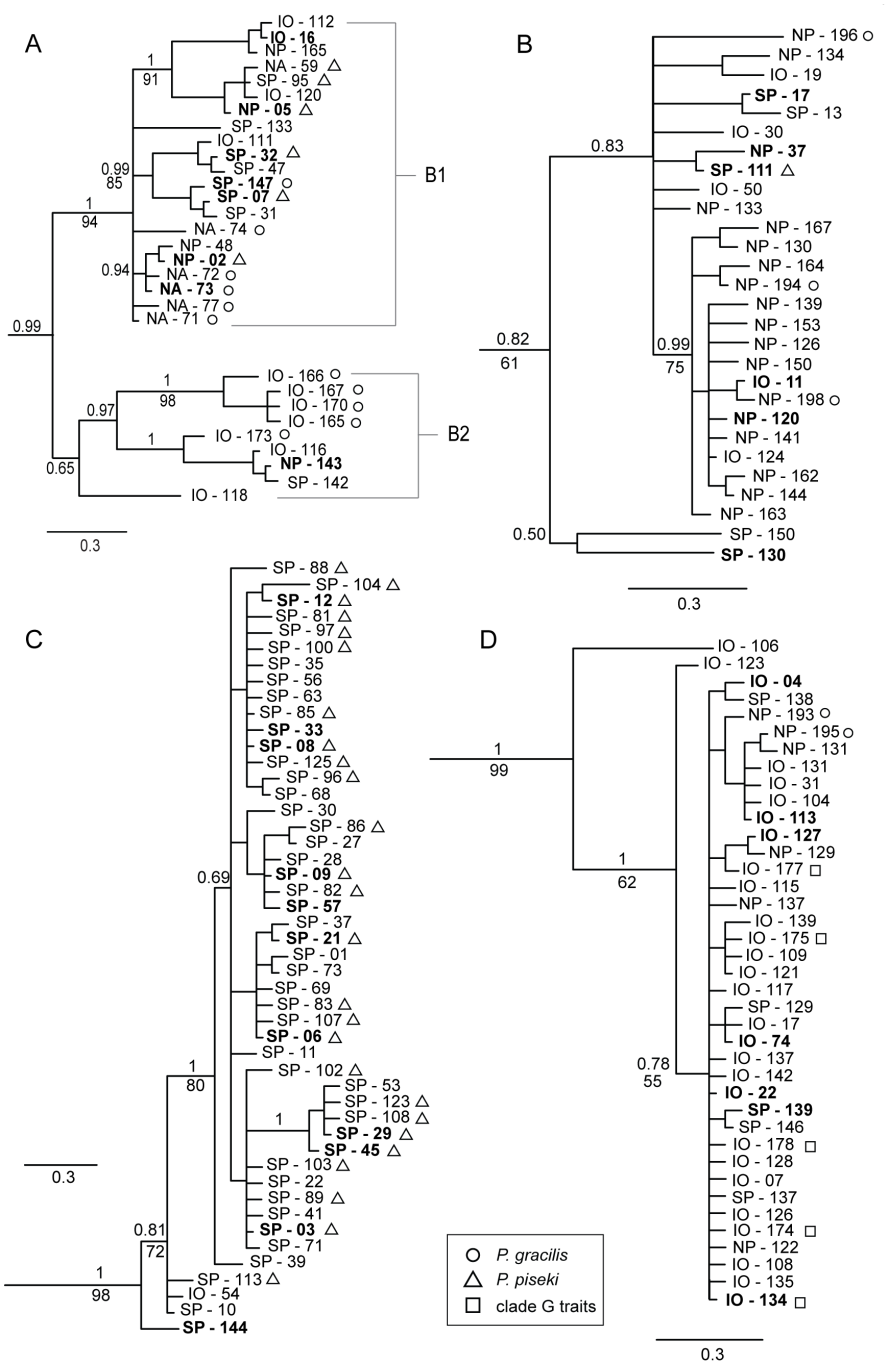
Bayesian mtCOII phylogenies of four clades in the *P. piseki* – *P. gracilis* species complex. (A) *P. piseki – P. gracilis* clade B, with sub-groups B1 and B2 as shown, (B) *P. piseki – P. gracilis* clade F, (C) *P. piseki – P. gracilis* clade C, and (D) *P. piseki – P. gracilis* clade G. Bayesian posterior probability values are given above the branches, and bootstrap values from ML analysis are given below the branches, when that node was supported in both Bayesian and ML analyses. **Bold** text indicates haplotypes that were sampled more than once. The scale bar units are in substitutions per site. Sample identifiers begin with the ocean basin that individuals were sampled from, IO = Indian Ocean, NP = North Pacific, SP = South Pacific, NA = North Atlantic, SA = South Atlantic. Shape symbols indicate morphological traits, as defined in the legend.

Twelve genetically divergent mitochondrial clades were found within the *Pleuromamma piseki – P. gracilis* species complex (Fig. 2). All 12 of these clades were well supported in Bayesian phylogenetic analyses (posterior probabilities >0.70) and in most cases also by maximum likelihood methods, indicating reciprocal monophyly for nearly all of these clades (Figs. 2, 3, 4). Of these 12, the five dominant clades, A, B, C, F and G, contained the majority of the genetic diversity sampled in this study, as well as the majority of specimens (Fig. 2, colored clades; Figs. 3, 4). Evolutionary divergence between these five dominant clades ranged from 6.3% (clades A & F; K2P-corrected distance) to 16.99% (clades C & G), with an average divergence of 11.44% between clades (Table 2). Clades C, E and L, in particular, were highly divergent from all other clades (>12%, Table 2). Within-clade divergences were an order of magnitude lower on average, and ranged between 0.24% (clade J) and 4.4% (clade B), with an average of 1.52% between haplotypes within the same clade (Table 2). The remaining seven clades, K, I, H, D, E, L and J, also were highly divergent from their closest relatives, for example clades K and D were placed within the basal polytomy for the species complex and were 6.75–17.11% divergent from all other groups (Fig. 2, Table 2). However, these clades were rare in our material, and we sampled only 1–13 individuals from each of these clades (1–5 haplotypes). For example, the unique sequence that constitutes clade L was sampled in only a single animal (Indian Ocean), but was 15.7% divergent from clade C, its closest relative (Table 2). Clade K also was represented by a single haplotype that was sampled in 4 animals collected in the subtropical North Pacific (2 locations, Table 1).

**Table 2:** Evolutionary divergence between major clades of the *P. piseki–P. gracilis* species complex.

	A1	A2	B1	B2	C	D	E	F	G	H	I	J	K	L
**A1**	0.97													
**A2**	3.0	1.29												
**B1**	9.2	8.1	2.59											
**B2**	9.3	8.5	6.5	4.2										
**C**	15.2	15.5	14.2	14.4	1.19									
**D**	8.7	7.6	10.5	9.5	15.8	2.9								
**E**	13.3	13.3	12.9	12.4	15.8	14.3	0.49							
**F**	6.6	5.8	9.5	9.3	15.4	8.3	13.5	2.28						
**G**	8.4	8.1	10.3	11.0	17.0	10.4	15.0	9.2	0.82					
**H**	6.6	5.4	9.4	10.1	16.2	8.1	14.3	6.5	6.1	0.36				
**I**	7.0	5.5	9.2	9.6	16.7	8.6	14.7	7.0	8.0	5.9	0.89			
**J**	8.1	7.9	9.1	9.0	15.9	8.3	12.8	7.1	10.4	9.1	8.6	0.24		
**K**	7.9	6.8	10.4	10.2	17.1	10.0	14.8	8.1	10.4	7.7	8.6	9.8	N/A	
**L**	14.5	14.9	13.3	13.4	15.7	16.3	16.3	14.7	16.1	15.3	16.4	15.7	16.6	N/A

Numbers along the diagonal represent the within-clade divergence for each clade. The analysis included 260 unique mtCOII haplotypes. Clades A–L are defined in figure 2. Values are given in percentages. N/A = not applicable (single haplotype found).

Major clades A and B both contained well-supported sub-groups (Figs. 3, 4A). In clade A, four sub-groups were well supported with Bayesian posterior probability values of >0.80 (Fig. 3A). The largest subgroup within clade A, referred to as A1 (Fig. 3), was genetically more divergent from the other sub-groups in clade A (2.5%–3.3%) than were the other three sub-groups (0.85%–1.5%). Therefore, these latter three groups were considered together as sub-group A2 (Fig. 3). The evolutionary divergence between sub-groups A1 and A2 was 3.04% (Table 2). Clades A1 and A2 were composed of 160 and 44 specimens, respectively. Clade B was composed of two well-supported sub-groups (Fig. 4A). Group B1 had high phylogenetic support (1.0 posterior probability, Fig. 4), while sub-group B2 was not as well supported due to the outlier sample IO-118 (0.65 posterior probability, Fig. 4). The posterior probability of this sub-group increased to 0.97 when this haplotype was excluded. Sub-group B2 had the highest within-group divergence in comparison to all other clades and sub-groups (4.2%; Table 2); even if the outlier sequence IO-118 was excluded, the within-group divergence was still the highest at 3.8%. Divergence between B1 and B2 was 6.46% (Table 2). The haplotypes in clades B1 and B2 were sampled in 58 and 10 specimens, respectively.

The topology of the Bayesian mtCOII tree for the whole species complex was a basal polytomy, with six groups ranging from low to modest support (Fig. 2). One well supported branch contained clades I, G, and H (0.98 posterior probability), with animals sampled in the tropical and eastern Pacific as well as the Indian Ocean (Table 1). A second group with modest support included clades E, C, L, and B (0.74 posterior probability); Bayesian analyses provided some support for placement of clade J within this group (0.63 posterior probability), but this node was not supported in ML analyses.

### Morphological Characters and Species Boundaries

The described species *P. piseki* and *P. gracilis* did not form monophyletic groups, rather, specimens with morphological traits characteristic of each described species were distributed throughout the phylogeny, sometimes co-occurring within well-resolved clades. Specimens identified as *P. piseki*, based primarily on the shape and size of the genital pigment spot and presence of the groove on the left side of the genital double-somite (ventral view, diagnostic trait), were placed with high support in clades A, B, C, F, and J (Figs. 2, 3, 4). Although clades A, C and J contained only animals identified as *P. piseki*, clades B and F each contained a mixture of specimens with morphological characters typical for *P. piseki* and *P. gracilis* (Figs. 2, 3, 4). *Pleuromamma gracilis* animals were placed in clades E, B, F, and G, with clade E and sub-group B2 the only clades that had exclusively *P. gracilis*-like animals. These results suggest that the morphological characters on the genital double-somite that are currently used to identify *P. piseki* and *P. gracilis* are incongruent with the mtDNA data.

**Figure 5:**
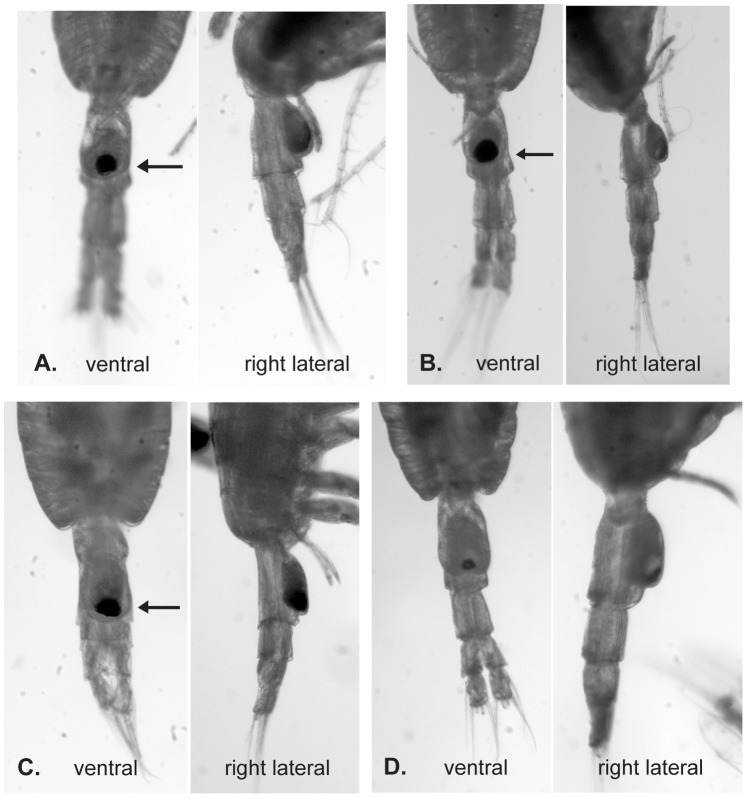
Digital images of the posterior prosome and urosome of *P. piseki* – *P. gracilis* animals from four genetic clades. Animals from (A) clade A, (B) clade C, (C) clade G, and (D) clade E are shown in ventral and right lateral views. By traditional morphological criteria, animals in (A), (B), and (C) would be identified as *P. piseki* and the animal in (D) would be identified as *P. gracilis*. Note that clade A and C both have a large black pigment spot at the copulatory pore, as well as a marked groove on the left side of the genital double-somite near the posterior margin (marked by an arrow), which was included in the original species description for *P. piseki* (Farran 1929). Clade G (panel C) also has a large black spot at the copulatory pore, but the spot is located at the posterior edge of the genital boss, and appears as a right-left oval rather than round in shape. Animals from this clade apparently lack the marked groove on the left side of the genital double-somite (absence visible at the black arrow). Finally, animals in clades E and B have genital characters that appear ‘gracilian’ (*P. gracilis*-like), as shown in panel D on a clade E specimen.

Our preliminary morphological observations suggest that there may be traits that distinguish the genetic clades. Clade E had significantly larger median prosome length in comparison to all other clades, with the exception of Clade G (Kruskal-Wallis ANOVA, *P*<0.05, followed by multiple comparisons). Across all clades, prosome length ranged from 1.17 to 1.57 mm, with a global average of 1.32 mm (Table 3). Mean prosome length in clade E was 1.47 mm (Table 3). Preliminary qualitative observations made on digital images of animals from four clades (A, C, G, E) also suggest that there may be differentiation between genetic clades in characters associated with the genital double-somite (Fig. 5). Variation was noted in the shape and location of the pigment spot on the genital somite (in ventral view), the shape of the genital boss in lateral view, and the presence/absence of the marked groove on the left side of the genital double-somite (ventral view; Fig. 5). Clades A and C appeared to be very similar morphologically, and both had traits characteristic of *P. piseki.* In these clades, the pigment spot at the copulatory pore was large and round, and both had a marked groove on the left side of the genital double-somite at the posterior margin that is a key character for identification of *P. piseki* [57]. Our initial observations suggest that Clade G may be distinct from other clades with *P. piseki*-like characters. Clade G animals had a large genital pigment spot, as in clades A and C, but it was located very distally on the genital boss (at the margin) and was distinctly oval, oriented left-right, rather than round in shape. Animals in this clade also lacked the characteristic groove on the left side of the genital double-somite (Fig. 5). The genital boss in right lateral view also appeared more elongate and less rounded than in Clades A and C. Finally, clade E was distinctly ‘gracilian’ in genital characters, with the traits visible in figure 5 largely as described for *P. gracilis*. The shape of the genital double-somite appeared as an up-side-down-heart in ventral view and in right lateral view the genital boss was quite thick even towards the anterior end: both traits were illustrated by Steuer [42] for his Forma ‘maxima’. Finally, the location of the pigment spot on the lateral prosome was invariant, and occurred on the right side in all animals.

**Table 3:** Summary statistics of prosome lengths (PL) measured on genotyped *P. piseki - P. gracilis* specimens.

Clade	N	Mean PL	Std Dev PL	Max PL	Min PL
		(mm)	(mm)	(mm)	(mm)
A	203	1.32	0.07	1.57	1.20
B	68	1.33	0.08	1.50	1.20
C	89	1.32	0.04	1.43	1.20
E	12	1.47*	0.11	1.60	1.30
F	42	1.30	0.04	1.40	1.20
G	69	1.34	0.04	1.43	1.23
I	6	1.23	0.08	1.37	1.17
J	9	1.29	0.03	1.33	1.23

Clades are defined in Fig. 2.

* clade E has significantly larger median PL compared to all clades except G (Kruskal-Wallis, multiple comparisons, *P*<0.05).

### Functional mtCOII Genes

**Figure 6:**
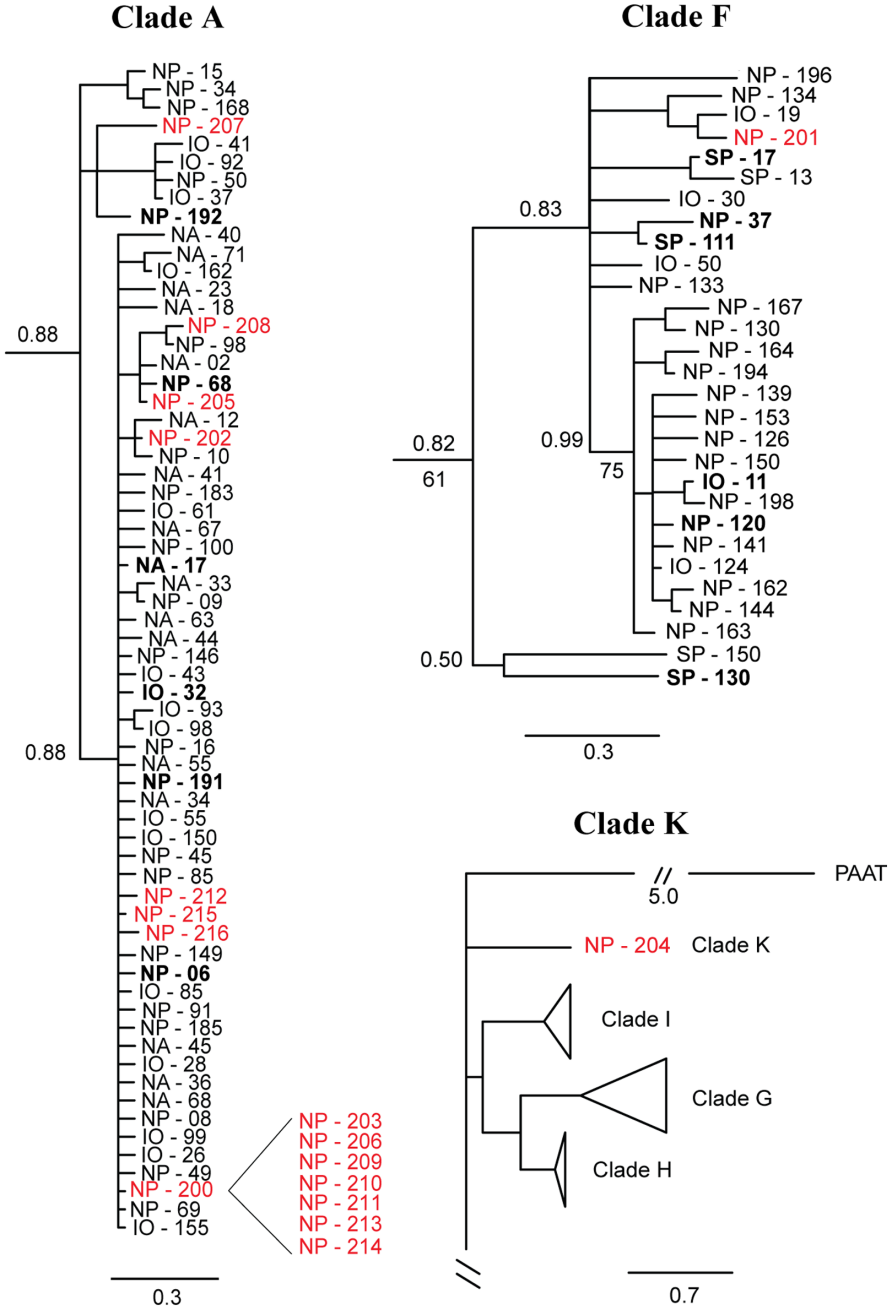
Placement of 17 cDNA mtCOII sequences in the Bayesian phylogeny of *P. piseki – gracilis* clades A, F, and K. cDNA sequences are marked in red font, and **bold** indicates haplotypes that were sampled more than once. The scale bar units are in substitutions per site. Relationships among the major clades are shown in figure 2.

cDNA and gDNA sequences obtained from the same animals using primer sets PLPICOIIFE – PLPICOIIRC (cDNA) and PLPICOIIFC – PLPIATP8R1 (gDNA) matched in all 17 specimens, demonstrating that the primer set used to obtain data for the phylogeny, PLPICOIIFC – PLPIATP8R1, consistently amplified the functional mtDNA gene copy. The majority of these specimens belonged to clade A (8 haplotypes, 15 specimens), one was placed in clade F, and one in clade K, confirming that all three of these clades represent real taxonomic entities within the *P. piseki* species complex and are not NUMT clades (Fig. 6). Additional evidence that the phylogeny primers were targeting mtDNA came from the presence of a stop codon at the end of the mtCOII gene in sequences generated from direct sequencing (1 exception, not included).

In a small number of PCRs (7%), the phylogeny PCR primer pair (PLPICOIIFC and PLPIATP8R1) resulted in double-banded products that were apparent on agarose gels and these products were not sequenced. In order to determine clade membership of specimens yielding multiple products, we selected eight of these samples and amplified a 376-bp PCR product (within mtCOII) using the forward phylogeny primer PLPICOIIFC and a reverse primer that targeted the 3′ end of mtCOII. Phylogenetic analysis of these sequences placed all eight in sub-group B2 (Fig. S3; [75]). Multiple NUMT sequences were found in some animals from which PCR products were cloned, and non-functional gene copies were identifiable by high rates of divergence and long branch lengths (Text S1, Fig. S3).

### Geographic Structure

**Figure 7:**
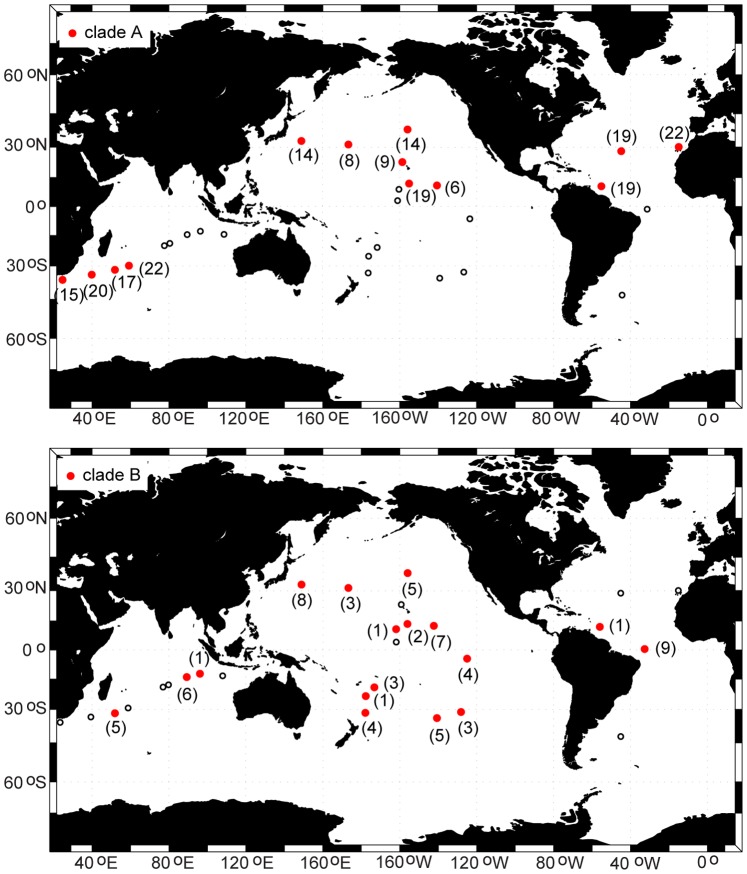
Distribution map for cosmopolitan clades A (top panel) and B (bottom panel). The number of specimens (n) for each site is given in parentheses beside the symbol. Small, open circles indicate sample locations that did not include any specimens of this clade (absence).

**Figure 8:**
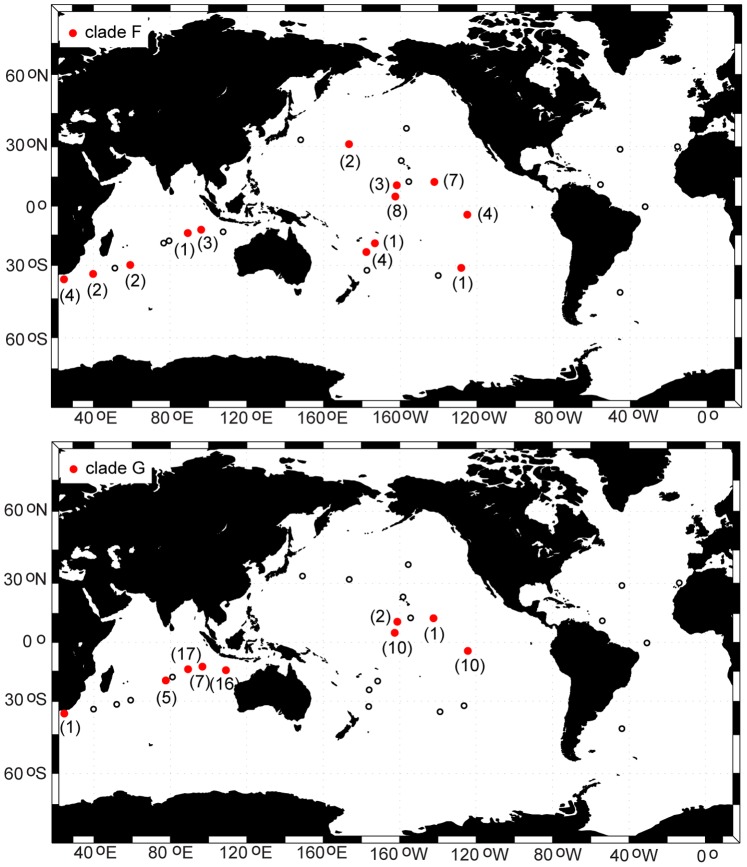
Distribution map for Indo-Pacific clades F (top panel) and G (bottom panel). The number of specimens (n) for each site is given in parentheses beside the symbol. Small, open circles indicate sample locations that did not include any specimens of this clade (absence).

Clades in the *P. piseki – P. gracilis* species complex ranged from being circumglobal in distribution to occurring at only a single site. Clades A and B were cosmopolitan, and occurred broadly throughout subtropical and tropical waters in all three major ocean basins (Fig. 7). The majority of specimens sampled in the North Atlantic were from clade A (3 sites), and this clade also dominated our material in the North Pacific (6 sites) and western Indian Ocean (4 sites; Fig. 7). Sample sizes for clade A specimens ranged between 6 and 22 individuals, with an average of 15 specimens per site (Table 1). Clade A was conspicuously absent from the South Pacific, though sample sizes were quite high there, and this clade also was apparently absent from the South Atlantic. Clade B occurred throughout the Pacific Ocean in subtropical and tropical waters, and also was present in the North Atlantic and Indian Oceans (Fig. 7). Clades F and G had Indo-Pacific distributions, and both genetic clades were absent from the Atlantic Ocean (Fig. 8). Clade G appeared to be more restricted to tropical and equatorial waters than clade F, which also occurred in subtropical waters of both the Pacific and Indian Oceans. The majority of clade C specimens were found in the South Pacific (Fig. S1; Table 1). Clade E was found exclusively in the southern transition zone in the Indian, Pacific, and Atlantic Oceans (Fig. S1). Clade J occurred in the Indian, South Pacific, and North Atlantic Oceans, although it appeared to be quite rare throughout its global range (Fig. S2). Only a few specimens were sampled from each of the remaining 5 clades, and they were located either in the North Pacific (clades D, K), Indian Ocean (clades I, L), or in the South Pacific near the equator (clade H, Figs. S1 & S2). A contingency analysis confirmed that the distribution and frequency of major clades among ocean basins was non-random (*χ^2^* = 1127; *P*<0.0001).

In the hierarchical AMOVA for clade A, a significant fraction of the variance was related to structure among ocean basins (Table S1, 10.5%, *P*<0.0001). There was no significant variation explained by the among localities component, consistent with pairwise θ_ST_ comparisons among all sampling sites (Table S2). However, some significant variation was seen within the North Pacific, between ACEASIA-08 and two other North Pacific locations: ACEASIA-14 (θ_ST_ = 0.15, *P*<0.05) and S230-037 (θ_ST = _0.10, *P*<0.05, Table S2). Pairwise θ_ST_ values between the Indian and North Pacific Oceans ranged between 0 and 0.23, with an average of 0.10. Similar values were found between the North Pacific and North Atlantic Oceans (θ_ST_ range: 0.03–0.22, average –0.12). No population structure was observed between the Indian and North Atlantic Oceans (θ_ST_ range: −0.03–0.04, Table S2).

## Discussion

As described in the introduction, the systematics of the genus *Pleuromamma* has been treated as though it is well resolved, and females of these species are thought to be quite straightforward to identify. A substantial ecological literature exists on *Pleuromamma piseki* and *P. gracilis* that relies on the existing morphological taxonomy, with detailed reports of their horizontal and vertical distributions [43], [44], spatial and temporal patterns of abundance [45], [50], population structure [76], trophic ecology [48], and physiological condition [51], among other topics [77]. Notably, relatively little ecological niche separation was observed between these two described species in a number of studies (e.g., [48]). However, our genetic results suggest that these described species are part of a species complex, and consist of a mosaic of evolutionarily divergent mitochondrial lineages that are distributed, in many cases, sympatrically across diverse ocean environments. Further, our morphological observations, although preliminary, indicate that the morphological traits currently in use to identify these species do not map clearly onto genetic clades, and prior ecological studies may have included spectra of genetically distinct taxa within each of these nominal species. These observations provide one possible explanation for the lack of apparent ecological specialization of *P. gracilis* and *P. piseki* in prior reports. Our global-scale sampling suggests that these new cryptic lineages range from being common to rare, and have distinct biogeographic distributions that range from being cosmopolitan in subtropical-tropical waters to being restricted to particular water mass types (e.g., equatorial waters of the Indo-Pacific). Below, we first discuss the evidence for divergent genetic clades and their possible status as cryptic, undescribed species, and then review the biogeographic distributions of these new lineages.

### Cryptic Species?

Twelve evolutionarily divergent mitochondrial lineages were discovered within this *Pleuromamma* species complex that were well supported in both Bayesian and maximum likelihood mtCOII trees (Bayesian posterior probability values <0.7, Fig. 2), with unambiguous reciprocal monophyly seen among all 12 clades in the Bayesian gene tree. Six of these clades were supported with Bayesian posterior probability values of 0.99 or higher (clades C, E, G, H, I, L). Evolutionary divergence values between clades (5–17% K2P distance) were high compared to within-clade divergences (average –1.5%, Table 2), and were broadly comparable to values seen between copepod species in other groups, in particular for the most divergent clades (e.g., [34], [61], [78], [79]). For example, evolutionary divergences of >4–25% at mitochondrial protein-coding genes in several marine crustaceans have been considered to be evidence of the existence of cryptic species (e.g., [80]–[82]). Supporting patterns of divergence at nuclear loci or in geography and morphology provided additional support for the inference that these lineages were reproductively isolated. Emerging evidence from other planktonic calanoids also suggests that recently divergent species can be separated by as few as 6–7 bp substitutions at this mitochondrial gene region, with clear concordance found among results from nuclear microsatellite and mitochondrial markers [30]. We also validated several features of the topology of our mtCOII tree by comparing cDNA sequences of mtCOII derived from RNA transcripts with gDNA sequences of mtCOII from the same individuals. Matching sequences in all specimens confirmed the validity of clades A, K, and F, and based on sequence properties in all other clades, we infer that all 12 mtDNA clades reflect mitochondrial evolution and are unlikely to be NUMT clades. These results all suggest that the divergent mitochondrial lineages found within *P. piseki* and *P. gracilis* likely reflect cryptic species. However, alternative explanations are possible, and given the absence of data from additional nuclear markers in this system, our results based on mtCOII are preliminary.

A second possible explanation for the observation of deeply divergent mtDNA lineages is that they are present within a single interbreeding species (or two species, as described). The large population sizes of marine holoplankton would be expected to slow the lineage sorting process, reducing the probability or rate of loss of ancient lineages within species [37], [83]. A few neritic and/or benthic copepod species also have very divergent mitochondrial lineages that are capable of interbreeding in laboratory crosses (in some cases with lower F2 hybrid fitness, e.g. [84]–[86]) and these complexes are used as model systems for understanding the genomic basis of speciation (in transition between populations and species). Furthermore, a number of studies have shown discordance between mitochondrial and nuclear markers in detecting either species boundaries or population structure (e.g., [39], [87], [88]), and in combination, these studies all indicate caution in relying on one or few genetic markers for inferring the presence of distinct species. We recognize the complexity of accurately inferring species boundaries: Therefore, we hypothesize that the mtCOII lineages within the *Pleuromamma piseki* – *P. gracilis* species complex represent distinct species, and note that further work is required to validate the appropriate systematic position of these ancient mtDNA lineages. In the discussion below, we proceed with the expectation that many of these lineages are likely to be undescribed, cryptic species, while recognizing that this inference requires validation in subsequent work.

One of the most unexpected aspects of our results was the observation that the morphological characters on the genital double-somite that are currently in use to distinguish *P. piseki* and *P. gracilis* appeared to be relatively uninformative with regards to likely species boundaries. Animals with *P. piseki*-like characters were found within five clades, with only three of these exclusively containing animals with *P. piseki* morphological traits (clades A, J, C). The remaining two clades contained animals with both *P. piseki* and *P. gracilis* morphological characters (clades B, F), indicating a mixture of morphotypes within these clades. Comparable results were observed for *P. gracilis* animals in that they sometimes co-occurred with *P. piseki* within well-resolved phylogenetic clades (e.g., clade B). These animals were identified to species based primarily on traits associated with the genital double-somite (e.g., pigment spot near the copulatory pore, presence of a marked groove on the left side of the somite), and our results suggest that these taxonomic characters are likely insufficient for distinguishing true species in this group. This finding is uncommon for copepods. To our knowledge all prior examples of cryptic species complexes have found broad congruence with existing morphological species descriptions, but with greater diversity within each morphologically defined species (e.g., [34]). This result has significant implications for the existing literature on *P. piseki* and *P. gracilis*, as it suggests that it may not have been possible to accurately identify true species using the described morphological characters in prior work on these species.

Prior systematic and biogeographic studies in the genus *Pleuromamma* have provided some suggestion of unresolved diversity within the group. Notably, Steuer [42] described three distinct forms within *P. gracilis* s. l., Forma minima, Forma maxima and Forma Piseki. Forma Piseki is now recognized as the contemporary species *P. piseki* [55], whereas Steuer’s Forma have largely fallen out of use in the current literature. Our results suggest that there is in fact a larger-bodied species with *P. gracilis*-like morphological characters that inhabits the southern transition zone of all ocean basins (1.3–1.6 mm PL for adult females; Table 3, Fig. S1). This lineage, clade E in our material, is largely congruent with Steuer’s observations of Forma maxima, with the exception that we find no evidence that clade E is distributed outside of the southern transition zone region. The morphology of the genital boss of our clade E specimens (Fig. 5D) shows the up-side-down heart shape seen in ventral view for *P. gracilis* Forma maxima (Steuer 1932, his Textfig. 134), and is also thicker in right lateral view than as described for Forma minima (compare our figure 5D to Steuer Textfig. 134 and 135). Therefore, following more extensive examination of clade E specimens, it may be appropriate to elevate Forma maxima to species. We also anticipate that *Pleuromamma gracilis* s.s. will correspond to genetic clade B of this study, once a thorough systematic revision has been completed. Resolving the position of *P. borealis*, the only remaining described small-bodied species in the genus, is also a high priority for future work. Our preliminary morphological observations on genital characters suggest that these traits will continue to be informative within the genus (e.g., see Fig. 5C), although the specific traits currently in use as taxonomic characters may not be definitive for identification to species. The systematic identity of the name-bearing lineages for described species *P. piseki* and *P. gracilis* remains to be verified, and will be challenging given broad sympatry of many of the undescribed lineages in the North Atlantic. The type locality for *P. piseki* was not specifically identified, although its distribution was listed as the North Temperate and Tropical Atlantic from 29°10′N southwards as well as off New Zealand [57], and *P. gracilis* was first described from Messina in the Mediterranean Sea [89]. Five genetic clades co-occur in these areas (North Atlantic; A, B, C, E, J; Table 1). It is unclear which of these may correspond to the material originally examined for species descriptions. Finally, there may be additional, undetected diversity within this species complex: a number of the divergent lineages discovered here were found in only a few individuals, suggesting that the global ocean remains under-sampled.

### Biogeography of Cryptic Lineages

The novel genetic lineages within *P. piseki* and *P. gracilis* appear to be ecologically divergent, with distinct biogeographic distributions across varied pelagic habitats. Among the 12 genetic clades, some were common in our material, and occurred at nearly half the collection sites (e.g., clade A), while others were rare, and collected at only a single sampling site (e.g., clades H, I, and L). The biogeographic distributions of common lineages therefore can be interpreted from the present material, while rare clades remain poorly characterized in terms of their distributions across pelagic habitats. Four clades had circumglobal distributions (clades A, B, C, and J) and were broadly sympatric across subtropical and tropical waters, indicating a capacity to thrive across a range of pelagic habitat types from broadly eutrophic to oligotrophic systems (e.g., subtropical gyres, equatorial provinces). These clades also were the most numerous at many of our sampling sites (in particular clades A, B). Two clades, F and G, were Indo-Pacific in distribution, and occurred sympatrically with each other as well as with the cosmopolitan clades. Although clade F was quite widespread across oligotrophic and eutrophic waters in the Pacific and Indian Oceans, clade G appeared to be largely restricted to equatorial waters, a distributional type that is common to many other zooplankton species (e.g., *Clausocalanus minor*, [90], *Subeucalanus subcrassus*, [19], *Euphausia diomediae,* [91]). We also found that many of the divergent clades occurred in only one or two locations within the same ocean basin (e.g. North Pacific, clades D, K), and sometimes consisted of only a single individual from our material (e.g., clade L). These observations suggest that the global diversity within the small-bodied *Pleuromamma* species is likely under-sampled in this study. Finally, although the branching topology of our mtCOII phylogeny was not well resolved at most deeper nodes within the tree (Fig. 2), widespread clades do not appear to be close relatives to one another, and in the cases where sister relationships were well-supported (e.g., Clades C, L), a widespread, common clade was most closely related to a lineage that was rare or restricted in distribution.

## Conclusions

Discovery of cryptic species is common, in particular for marine invertebrate groups (e.g., [31], [92]). However, an inability to identify common, reproductively isolated species can impede understanding of ecosystem dynamics, making discovery and description of cryptic species complexes an important endeavor. *Pleuromamma* is the most ecologically significant genus of the diel vertical migratory (DVM) zooplankton assemblage, due to the high *in situ* abundance and global distribution of members of this genus, as well as their extensive vertical migrations, which result in active export of carbon and nitrogen from surface waters into the deep ocean. Members of this genus, including *P. gracilis* and *P. piseki*, have been reported as numerical dominants of the zooplankton assemblage in surface waters at night [45]. Results reported here demonstrate that extensive cryptic diversity exists within smaller members of this common copepod genus, with broad sympatry of >5 divergent mtDNA lineages in most regions of the global ocean. We hypothesize that many of these mtDNA lineages are undescribed, cryptic species, and call for formal validation of the systematic position of these lineages through further work on morphological and genetic variation in this species complex. These putative new species show evidence of ecological divergence, with distinct biogeographic distributions across water mass types. Formal description of these new species will be important to detecting long-term trends in abundance of the dominant species in marine zooplankton communities, resolving their population responses to climate forcing, and inferring their impact on biogeochemical cycling in the upper ocean.

## Supporting Information

Figure S1 Distribution map for clades C, D, I and E. The number of specimens (n) for each site is given in parentheses beside the symbol. Small, open circles indicate sample locations that did not include any specimens of these clades (absence). Colors and symbols for each clade as indicated in the legend.
(TIF)

Figure S2 Distribution map for clades J, K, L, and H. The number of specimens (n) for each site is given in parentheses beside the symbol. Small, open circles indicate sample locations that did not include any specimens of these clades (absence). Colors and symbols for each clade as indicated in the legend.
(TIF)

Figure S3 Bayesian phylogeny of *P. piseki – P. gracilis* clades B and F, incorporating sequences from cloning experiments and other problematic data from genomic DNA amplifications. (A) Bayesian phylogeny of clade B, (B) Bayesian phylogeny of clade F. Green text = sequences cloned from genomic DNA amplifications. Blue text = mtCOII amplifications (376-bp) with primers PLPICOIIFC & COIIR10 that were used to determine the clade membership of animals yielding multiple products in PCR. NUMTs (premature stop codon) are highlighted in grey. **Bold** text indicates haplotypes that were sampled more than once. The scale bar units are in substitutions per site.
(TIF)

Table S1 Population structure within clade A, across the Indian, North Pacific, and North Atlantic Oceans. Results from a hierarchical analysis of molecular variance (AMOVA).
(DOCX)

Table S2 Pairwise θ_ST_ values between sampling sites within clade A. Significant values (*P*-value <0.05) are shown in **bold**. The Q-value was <0.05 for all *P*-values <0.05. Indian Ocean sites = VANC-02, VANC-06, VANC-09, VANC-11; North Pacific sites = ASIA-14, ASIA-08, HOT, S226-48, S230-37, STAR-49; North Atlantic sites = TRAN, MP3-12, MP3-14.
(DOCX)

Text S1 Supplementary Text on characterizing NUMTs.
(DOCX)
