# Supplementary figures and images for: High Cryptic Diversity across the Global Range of the Migratory Planktonic Copepods Pleuromamma piseki and P. gracilis
Source: PLoS One. 2013 Oct 22;8(10):e77011. doi: 10.1371/journal.pone.0077011 (PMC3805563; doi:10.1371/journal.pone.0077011)

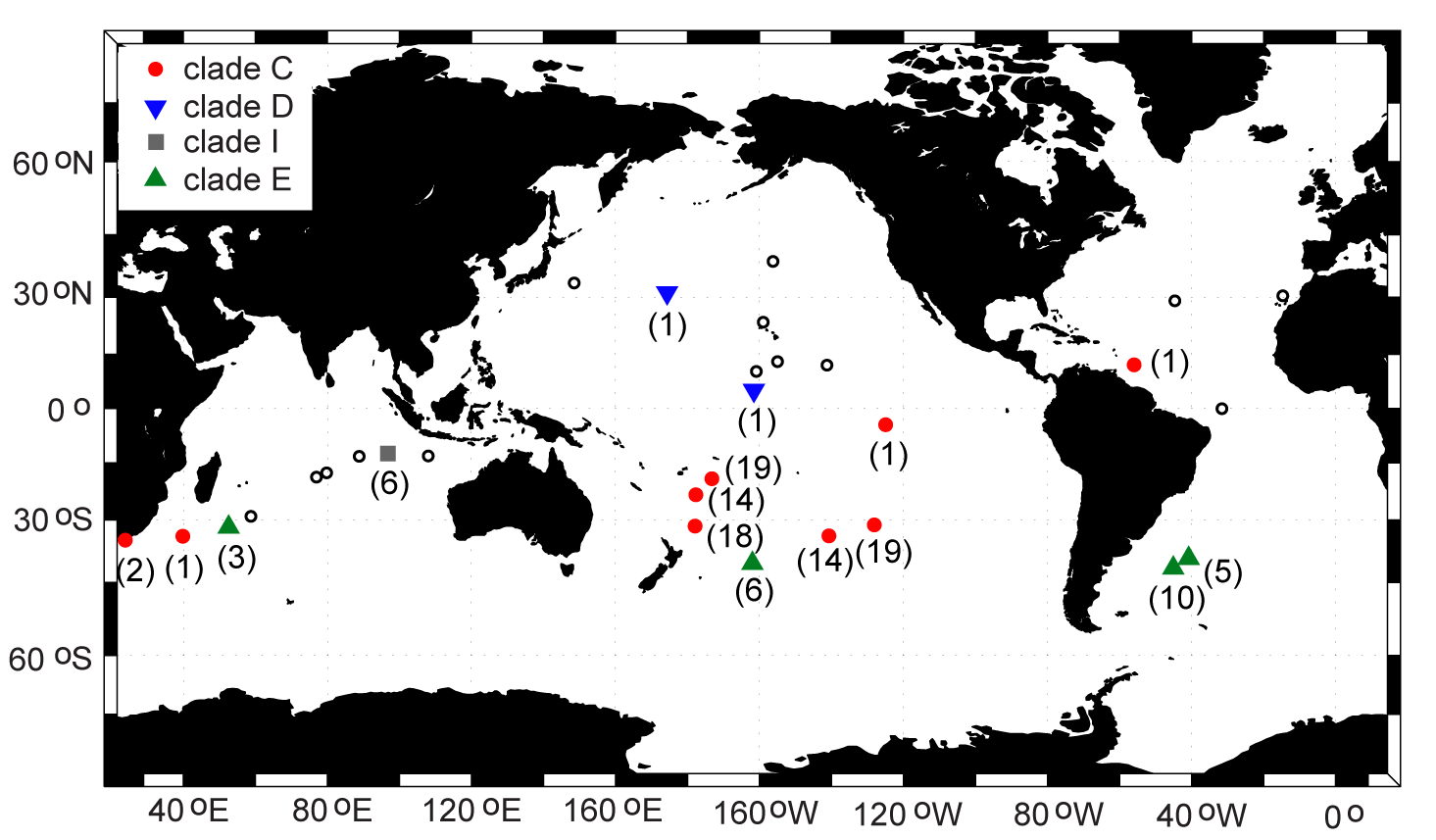

Supplement: Figure S1 — Distribution map for clades C, D, I and E. The number of specimens (n) for each site is given in parentheses beside the symbol. Small, open circles indicate sample locations that did not include any specimens of these clades (absence). Colors and symbols for each clade as indicated in the legend. (TIF) [file pone.0077011.s001.tif]

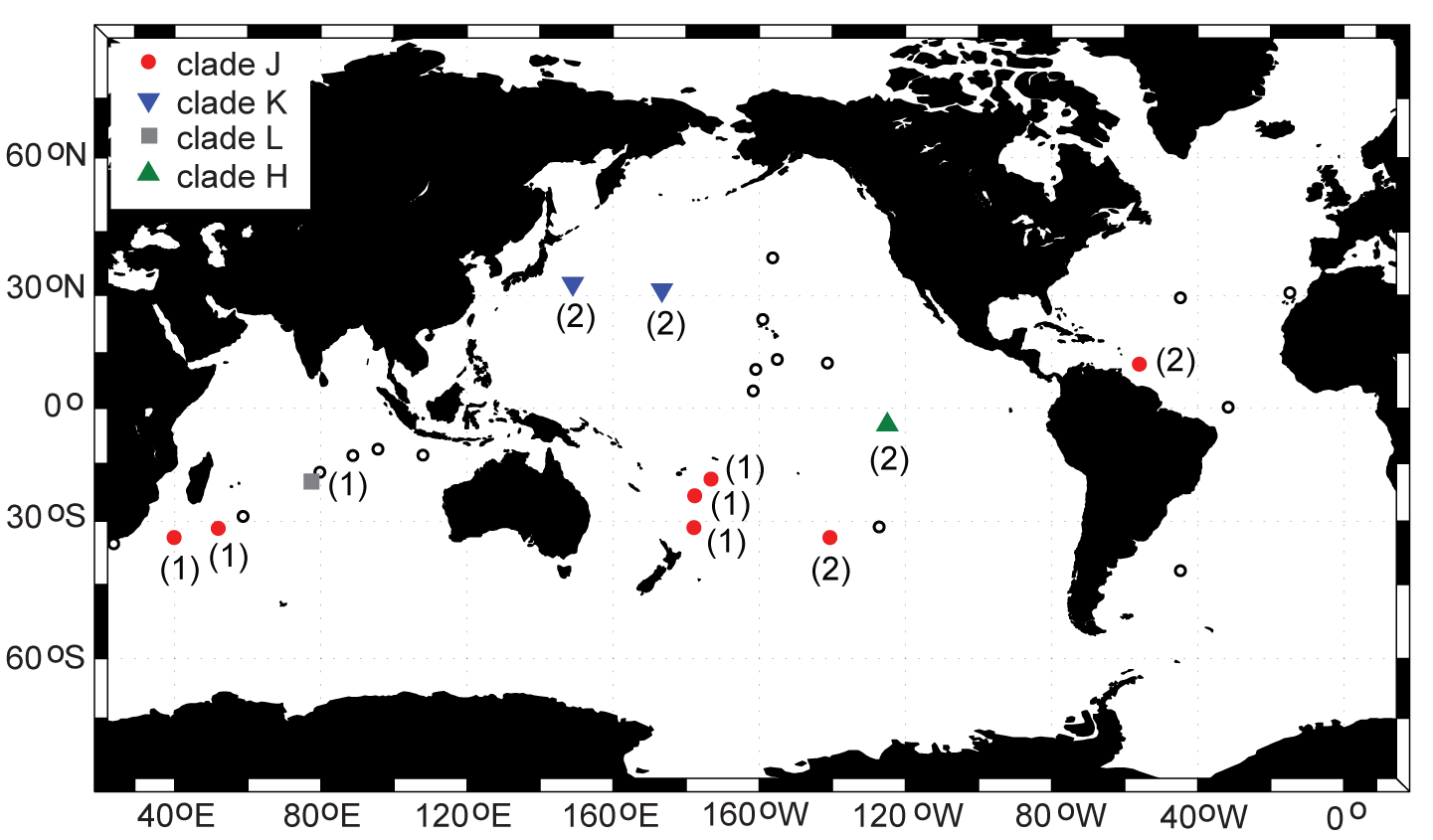

Supplement: Figure S2 — Distribution map for clades J, K, L, and H. The number of specimens (n) for each site is given in parentheses beside the symbol. Small, open circles indicate sample locations that did not include any specimens of these clades (absence). Colors and symbols for each clade as indicated in the legend. (TIF) [file pone.0077011.s002.tif]

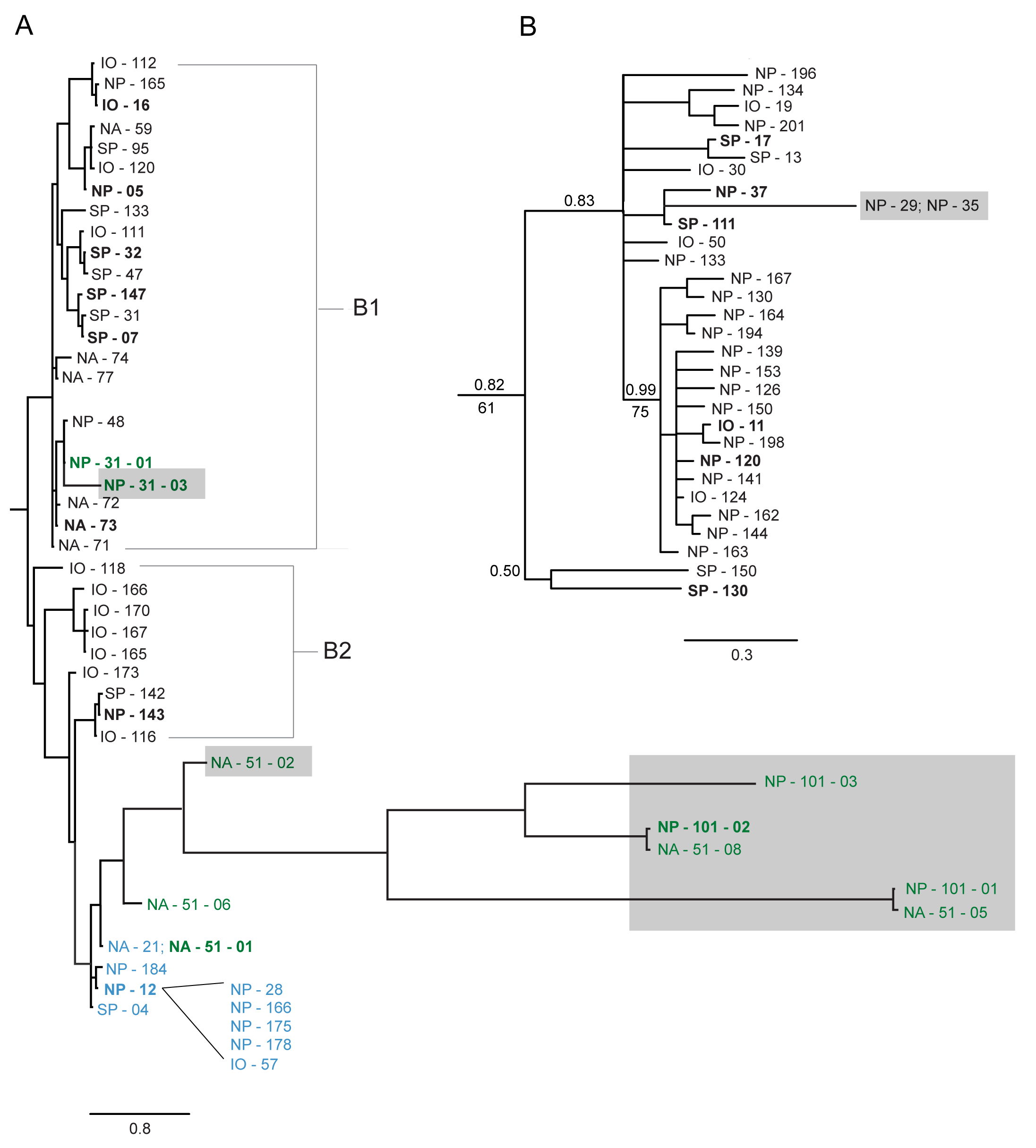

Supplement: Figure S3 — Bayesian phylogeny of P. piseki – P. gracilis clades B and F, incorporating sequences from cloning experiments and other problematic data from genomic DNA amplifications. (A) Bayesian phylogeny of clade B, (B) Bayesian phylogeny of clade F. Green text = sequences cloned from genomic DNA amplifications. Blue text = mtCOII amplifications (376-bp) with primers PLPICOIIFC & COIIR10 that were used to determine the clade membership of animals yielding multiple products in PCR. NUMTs (premature stop codon) are highlighted in grey. Bold text indicates haplotypes that were sampled more than once. The scale bar units are in substitutions per site. (TIF) [file pone.0077011.s003.tif]
