# Supplementary material for: High Cryptic Diversity across the Global Range of the Migratory Planktonic Copepods Pleuromamma piseki and P. gracilis
Source: PLoS One. 2013 Oct 22;8(10):e77011. doi: 10.1371/journal.pone.0077011 (PMC3805563; doi:10.1371/journal.pone.0077011)
Supplement: Table S1 — Population structure within clade A, across the Indian, North Pacific, and North Atlantic Oceans. Results from a hierarchical analysis of molecular variance (AMOVA). (DOCX) [file pone.0077011.s004.docx]

**Supplementary Tables**

Table S1. Population structure within clade A, across the Indian, North Pacific, and North Atlantic Oceans. Results from a hierarchical analysis of molecular variance (AMOVA).

| Variation component | Degrees of Freedom | Sum of Squares | Variance Components | Percentage of Variation |
| --- | --- | --- | --- | --- |
| Among Ocean Basins | 2 | 59.963 | 0.41252 | 10.5 |
| Among Localities | 10 | 22.443 | -0.08967 | -2.28 |
| Within Populations | 191 | 688.437 | 3.60438 | 91.78 |
