# Supplementary material for: High Cryptic Diversity across the Global Range of the Migratory Planktonic Copepods Pleuromamma piseki and P. gracilis
Source: PLoS One. 2013 Oct 22;8(10):e77011. doi: 10.1371/journal.pone.0077011 (PMC3805563; doi:10.1371/journal.pone.0077011)
Supplement: Table S2 — Pairwise θST values between sampling sites within clade A. Significant values (P-value <0.05) are shown in bold. The Q-value was <0.05 for all P-values <0.05. Indian Ocean sites = VANC-02, VANC-06, VANC-09, VANC-11; North Pacific sites = ASIA-14, ASIA-08, HOT, S226-48, S230-37, STAR-49; North Atlantic sites = TRAN, MP3-12, MP3-14. (DOCX) [file pone.0077011.s005.docx]

Table S2. Pairwise θ_ST_ values between sampling sites within clade A.

|  | VANC-02 | VANC-06 | VANC-09 | VANC-11 | ASIA-14 | ASIA-08 | HOT | S226-48 | S230-037 | STAR-49 | TRAN | MP3-12 | MP3-14 |
| --- | --- | --- | --- | --- | --- | --- | --- | --- | --- | --- | --- | --- | --- |
| VANC-02 | *** |  |  |  |  |  |  |  |  |  |  |  |  |
| VANC-06 | -0.04 | *** |  |  |  |  |  |  |  |  |  |  |  |
| VANC-09 | -0.04 | -0.03 | *** |  |  |  |  |  |  |  |  |  |  |
| VANC-11 | 0.00 | 0.00 | -0.03 | *** |  |  |  |  |  |  |  |  |  |
| ASIA-14 | **0.23** | **0.19** | **0.15** | **0.09** | *** |  |  |  |  |  |  |  |  |
| ASIA-08 | 0.10 | 0.08 | 0.07 | 0.02 | **0.15** | *** |  |  |  |  |  |  |  |
| HOT | 0.12 | 0.10 | 0.06 | 0.02 | 0.04 | -0.04 | *** |  |  |  |  |  |  |
| S226-48 | **0.17** | **0.15** | **0.10** | 0.05 | 0.01 | 0.00 | -0.03 | *** |  |  |  |  |  |
| S230-37 | **0.20** | **0.17** | **0.12** | 0.07 | -0.01 | **0.10** | 0.00 | 0.00 | *** |  |  |  |  |
| STAR-49 | 0.10 | 0.08 | 0.05 | 0.00 | 0.05 | -0.07 | -0.06 | -0.09 | 0.00 | *** |  |  |  |
| TRAN | -0.01 | 0.00 | -0.01 | 0.03 | **0.16** | 0.08 | 0.09 | **0.13** | **0.15** | 0.07 | *** |  |  |
| MP3-12 | -0.02 | -0.01 | -0.03 | 0.00 | **0.12** | 0.04 | 0.05 | **0.09** | **0.11** | 0.03 | -0.02 | *** |  |
| MP3-14 | -0.02 | -0.01 | 0.00 | 0.04 | **0.22** | 0.11 | **0.13** | **0.18** | **0.20** | 0.11 | -0.03 | -0.02 | *** |

Significant values (*P*-value < 0.05) are shown in **bold**. The Q-value was < 0.05 for all *P*-values < 0.05. Indian Ocean sites = VANC-02, VANC-06, VANC-09, VANC-11; North Pacific sites = ASIA-14, ASIA-08, HOT, S226-48, S230-37, STAR-49; North Atlantic sites = TRAN, MP3-12, MP3-14.
