# Supplementary material for: High Cryptic Diversity across the Global Range of the Migratory Planktonic Copepods Pleuromamma piseki and P. gracilis
Source: PLoS One. 2013 Oct 22;8(10):e77011. doi: 10.1371/journal.pone.0077011 (PMC3805563; doi:10.1371/journal.pone.0077011)
Supplement: Text S1 — Supplementary Text on characterizing NUMTs. (DOCX) [file pone.0077011.s006.docx]

**Supplementary Text on characterizing NUMTs**

Halbert K, Goetze E, Carlon DB (2013) High Cryptic Diversity across the Global Range of the Migratory Planktonic Copepods *Pleuromamma piseki* and *P. gracilis. PLOS One*

Legend: Brief description of the materials, methods and results of research conducted to characterize NUMTs in *P. piseki – P. gracilis*. (A) Materials & Methods used in cloning experiments, (B) Results from sequences obtained by cloning PCR products.

(A) Materials & Methods

To sequence and characterize potential NUMTs present in *Pleuromamma piski* and *P. gracilis*, we chose three individuals in which we clearly amplified different sized fragments and cloned them. PCR was performed in 40 µl reactions using the same parameters and primers as described above. PCR products were extracted from 1.5% agarose gels using the QIAquick Gel extraction Kit (Qiagen) following the manufacturer’s protocol. Cloning was performed using the TOPO TA Cloning Kit with Mach1 – T1 Chemically Competent *E. coli* cells (Invitrogen) following the manufacturer’s protocol. For each reaction, two agar plates were prepared with 10 µl and 30 µl of transformed cells plated on each plate. DNA was isolated from colonies using a Qiagen Plasmid Prep Kit following the manufacture’s protocol, and sequenced using M13 primers. DNA sequence was obtained from nine colonies each for two specimens, and seven colonies were sequenced from the third specimen.

(B) Results

Cloning of three specimens that yielded different sized PCR products yielded both functional mt gene copies and NUMTs as determined by the absence of a stop codon. In two of these individuals, we isolated and sequenced >2 unique NUMT alleles. Phylogenetic analysis placed all NUMT sequences in Clade B (Fig. S3, Halbert 2013), and NUMTs were easily identifiable by high rates of divergence and long branch lengths. NUMT sequences are available in GenBank under accession numbers KF546313 - KF546319.
